# Supplementary material for: Encouraging and Reinforcing Safe Breastfeeding Practices during the COVID-19 Pandemic
Source: Int J Environ Res Public Health. 2023 Jan 18;20(3):1756. doi: 10.3390/ijerph20031756 (PMC9914864; doi:10.3390/ijerph20031756)
Supplement: Supplementary file 1 [file ijerph-20-01756-s001.zip › ijerph-2024947-supplementary.pdf]

BREASTFEEDING SAFELY DURING COVID-19 PANDEMIC

IT IS SAFE TO BREASTFEED DURING THE COVID-19 PANDEMIC.

Breastmilk provides antibodies that help protect babies against many infections, including the Coronavirus. These are precautions you should take to protect yourself and your baby from COVID-19.

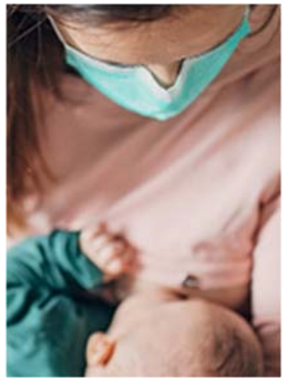

1). COVID-19 vaccination is safe for pregnant and breastfeeding mothers.

2). Stay home as much as possible, and limit outings with your baby.

3). Maintain 6 feet social distancing with other people in public places.

4). Sanitize or wash your hands with soap and water frequently and before you hold or breastfeed your baby.

5). Consider wearing a facemask while feeding your baby at the breast or from a feeding bottle.

6). Restrict visitors to your home and around your baby.

7). Keep the breast pump, milk storage containers and feeding utensils clean all the time as usual.

YOUR HEALTH CARE PROVIDERS ARE THERE FOR YOU. ALWAYS ASK THEM FOR ADVICE.

Breastfeed your baby from birth until 2 years old!

BREASTFEEDING SAFELY DURING COVID-19 PANDEMIC

IT IS SAFE TO BREASTFEED DURING THE COVID-19 PANDEMIC.

Breastmilk provides antibodies that help protect babies against many infections, including the Coronavirus. These are precautions you should take to protect yourself and your baby from COVID-19.

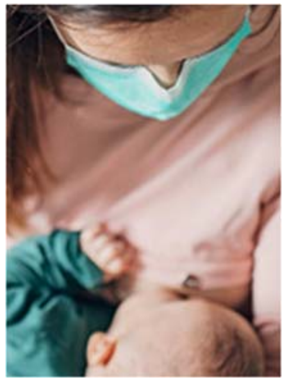

1). COVID-19 vaccination is safe for pregnant and breastfeeding mothers.

2). Stay home as much as possible, and limit outings with your baby.

3). Maintain 6 feet social distancing with other people in public places.

4). Sanitize or wash your hands with soap and water frequently and before you hold or breastfeed your baby.

5). Consider wearing a facemask while feeding your baby at the breast or from a feeding bottle.

6). Restrict visitors to your home and around your baby.

7). Keep the breast pump, milk storage containers and feeding utensils clean all the time as usual.

YOUR HEALTH CARE PROVIDERS ARE THERE FOR YOU. ALWAYS ASK THEM FOR ADVICE.

Breastfeed your baby from birth until 2 years old!

BREASTFEEDING SAFELY DURING COVID-19 PANDEMIC

IT IS SAFE TO BREASTFEED DURING THE COVID-19 PANDEMIC.

Breastmilk provides antibodies that help protect babies against many infections, including the Coronavirus. These are precautions you should take to protect yourself and your baby from COVID-19.

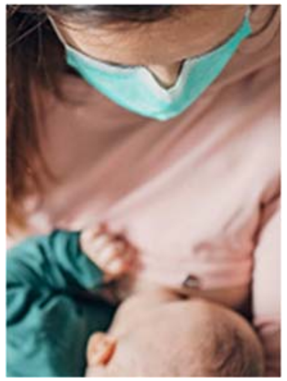

1). COVID-19 vaccination is safe for pregnant and breastfeeding mothers.

2). Stay home as much as possible, and limit outings with your baby.

3). Maintain 6 feet social distancing with other people in public places.

4). Sanitize or wash your hands with soap and water frequently and before you hold or breastfeed your baby.

5). Consider wearing a facemask while feeding your baby at the breast or from a feeding bottle.

6). Restrict visitors to your home and around your baby.

7). Keep the breast pump, milk storage containers and feeding utensils clean all the time as usual.

YOUR HEALTH CARE PROVIDERS ARE THERE FOR YOU. AL-WAYS ASK THEM FOR ADVICE.

Breastfeed your baby from birth until 2 years old!

BREASTFEEDING TIPS

1. Let your doctor (OB) know you plan to breastfeed your baby.
2. Tell your OB you wish to initiate breastfeeding within one hour of birth so that your baby gets colostrum.
3. Do not use a breast pump in the days after delivery. The breast produces only very little quantities of colostrum that cannot be extracted with a pump.

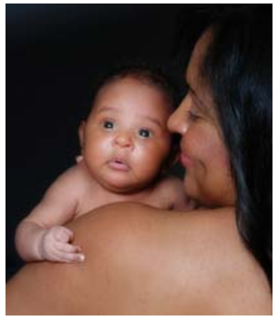

4. Put your baby to the breast several times by day and night: 10 times or more everyday to stimulate breastmilk production.
5. Stay at home with your baby for at least 2 months as caring for a newborn is a full-time job.
6. Do not use a breast pump until white breastmilk flow is well established.
7. Consider using a breast pump once a day 2 months after delivery to start practicing storing breastmilk.
8. Ask your baby's father, family and friends to help you with house chores and grocery shopping. Doing so will allow you to focus on caring for your newborn.
9. It is wise to avoid activities and persons that divert your attention from your baby. Your baby needs your full attention.
10. Your baby will be content with the barest minimum. At least you can breastfeed first thing in the morning, last thing before going to bed, and once or twice during the night.

Exclusive Breastfeeding from birth to 6 months old.

BREASTFEEDING TIPS

1. Let your doctor (OB) know you plan to breastfeed your baby.
2. Tell your OB you wish to initiate breastfeeding within one hour of birth so that your baby gets colostrum.
3. Do not use a breast pump in the days after delivery. The breast produces only very little quantities of colostrum that cannot be extracted with a pump.

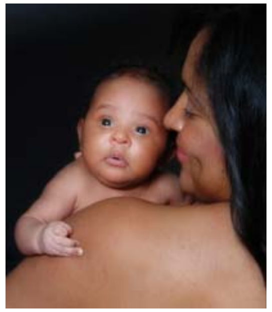

4. Put your baby to the breast several times by day and night: 10 times or more everyday to stimulate breastmilk production.
5. Stay at home with your baby for at least 2 months as caring for a newborn is a full-time job.
6. Do not use a breast pump until white breastmilk flow is well established.
7. Consider using a breast pump once a day 2 months after delivery to start practicing storing breastmilk.
8. Ask your baby's father, family and friends to help you with house chores and grocery shopping. Doing so will allow you to focus on caring for your newborn.
9. It is wise to avoid activities and persons that divert your attention from your baby. Your baby needs your full attention.
10. Your baby will be content with the barest minimum. At least you can breastfeed first thing in the morning, last thing before going to bed, and once or twice during the night.

Exclusive Breastfeeding from birth to 6 months old.

BREASTFEEDING TIPS

1. Let your doctor (OB) know you plan to breastfeed your baby.
2. Tell your OB you wish to initiate breastfeeding within one hour of birth so that your baby gets colostrum.
3. Do not use a breast pump in the days after delivery. The breast produces only very little quantities of colostrum that cannot be extracted with a pump.

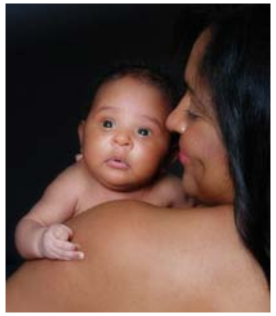

4. Put your baby to the breast several times by day and night: 10 times or more everyday to stimulate breastmilk production.
5. Stay at home with your baby for at least 2 months as caring for a newborn is a full-time job.
6. Do not use a breast pump until white breastmilk flow is well established.
7. Consider using a breast pump once a day 2 months after delivery to start practicing storing breastmilk.
8. Ask your baby's father, family and friends to help you with house chores and grocery shopping. Doing so will allow you to focus on caring for your newborn.
9. It is wise to avoid activities and persons that divert your attention from your baby. Your baby needs your full attention.
10. Your baby will be content with the barest minimum. At least you can breastfeed first thing in the morning, last thing before going to bed, and once or twice during the night.

Exclusive Breastfeeding from birth to 6 months old.

Approved by Meharry IRB
Expires on 10/06/2023

MEHARRY MEDICAL COLLEGE

No Cost Safe Breastfeeding Advice During COVID-19 Pandemic

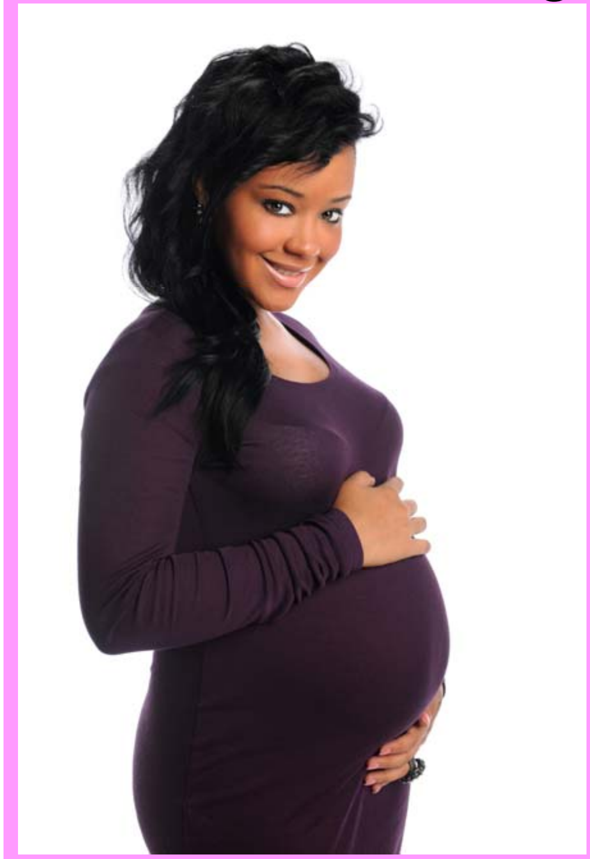

- Strategies to safely meet your breastfeeding goals.
- Learn to protect your baby from COVID-19 while breastfeeding
- Learn to prepare healthy homemade baby food.
- Request an appointment by phone or email to complete an infant feeding survey.
- Incentives: 3 Gift cards, Breastfeeding t-shirt, Food processor.

This project is housed at
Meharry Medical College, Nashville Tennessee.

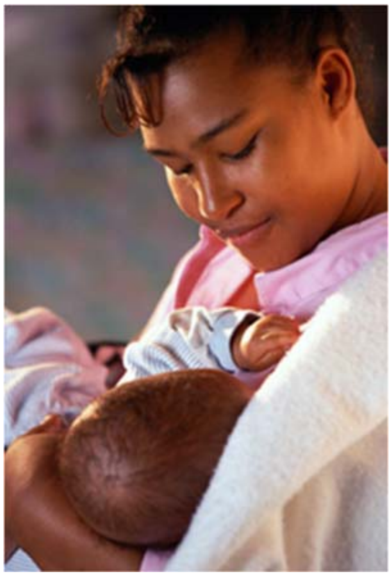

For more information contact:
COVID-19 Safe Breastfeeding Project
Tel: 615-327-5653   Email: fukoli@mmc.edu

Breastfeed Safely

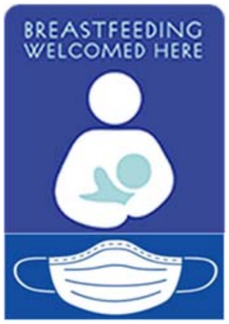

Meharry Medical College

Breastfeeding Promotion Program

To Enroll
Call: 615-327-5653
Email: fukoli@mmc.edu

Inviting Women in the 3rd Trimester.

Enroll and complete a 30-minute survey.
(Receive $20 Gift certificate)

15-minute follow-up survey 1 month after delivery.
(Receive $20 Gift certificate)

15-minute follow-up survey 4 months after delivery.
(Receive $20 Gift certificate)

Expect other gifts when you complete the program.
Nursing shirt, Food processor, Infant t-shirt.
